# Potential correlation between hemodynamic improvement and an immune-modulation effect in pediatric patients with septic shock treated with renal replacement therapy and CytoSorb^®^: an insight from the PedCyto study

**DOI:** 10.1186/s13054-024-04802-9

**Published:** 2024-01-17

**Authors:** Gabriella Bottari, Corrado Cecchetti, Carmela Serpe, David Grimaldi, Fabio S. Taccone

**Affiliations:** 1 Pediatric Intensive Care Unit, Bambino Gesù Children’s Hospital, IRCCS, Piazzale Sant’Onofrio 64, Rome, Italy; 2 Department of Intensive Care, Hopital Universitaire de Bruxelles (HUB), Université Libre de Bruxelles (ULB), Brussels, Belgium

Dear Editor,

Extracorporeal blood purification has been used in recent decades in sepsis, but its real efficacy is still debated. One of the beneficial effects suggested by authors for this adjuvant therapy is to restore immune-homeostasis in septic shock [1]. Because of the underlying dysregulated response to infection, extracorporeal therapies may help with an overwhelming cytokine production associated with fulminant septic shock and early death, but also for reducing the risk of developing immune-paralysis, which is correlated to late mortality [1, 2]. Among extracorporeal therapies, CytoSorb^®^ is one of the most investigated techniques in this field. CytoSorb^®^ is a cartridge made of copolymer beads intended for direct hemoadsorption that has shown a consistently good safety profile in published literature. It targets molecules in the 5–60 kDa range, which includes the molecular mass of several cytokines and inflammatory mediators.

We recently showed in a cohort of children with septic shock a significant decrease in Vasoactive Inotropic Score (VIS) and Pediatric Logistic Organ Dysfunction 2 (PELOD-2) score at 72 and 96 h from the start of CytoSorb^®^ therapy, compared to baseline [3]; the reductions observed were larger in the hemoadsorption group than a historical cohort of pediatric patients treated with only Continuous Renal Replacement Therapy (CRRT). Similarly, 28-day mortality was lower, although not significantly, in this group compared to the controls [3]. The median duration of hemoadsorption was 72 h (48–96 h) and each patient received extracorporeal therapy within 24 h from the onset of septic shock [3].

We also investigated if the hemodynamic improvement was associated with an immune-modulation effect of CytoSorb^®^ and CRRT in the same population (*n* = 17). Our final hypothesis was whether use of hemoadsorption, through control of the cytokine storm, could lead to less incidence of immune-dysfunction in this population. We measured the time course of Interleukin-IL-6, IL-10 and Tumor Necrosis Factor alpha (TNF-a) from baseline (*t*_0_ onset of hemoadsorption therapy) till 24 h after the end of treatment (*t*_+24h_). We also calculated the removal ratio (RR%) of cytokines between *t*_0_ and the end of hemoadsorption (*t*_end_), as = [Concentration at baseline (CB0)—Concentration at the end of the treatment (Cend)/CB0 × 100]. Changes in leukocyte count and in the class II major histocompatibility complex molecule (HLA)-DR on the surface of circulating monocytes were also measured, as a percentage and quantitative measurement with flow cytometry (HLA-DR MFI) at three monitoring timepoints of:* t*0 (D_0_), after 3 days (D_3_) and after 7 days (D_7_).

Figure 1 shows box plots for the time courses of IL-6, IL-10 and TNF-a at different timepoints. A significant reduction in IL-6 and IL-10, but not TNF-a, was observed over time. The RR% for IL-6 was 87% (IQR 76–96), for IL-10 66% (54–89) and for TNF-a 78% (54–96). No significant difference in the expression of HLA-DR antigen and in leukocytes count was observed over this time (Additional file 1: Fig. S1).

**Fig. 1:**
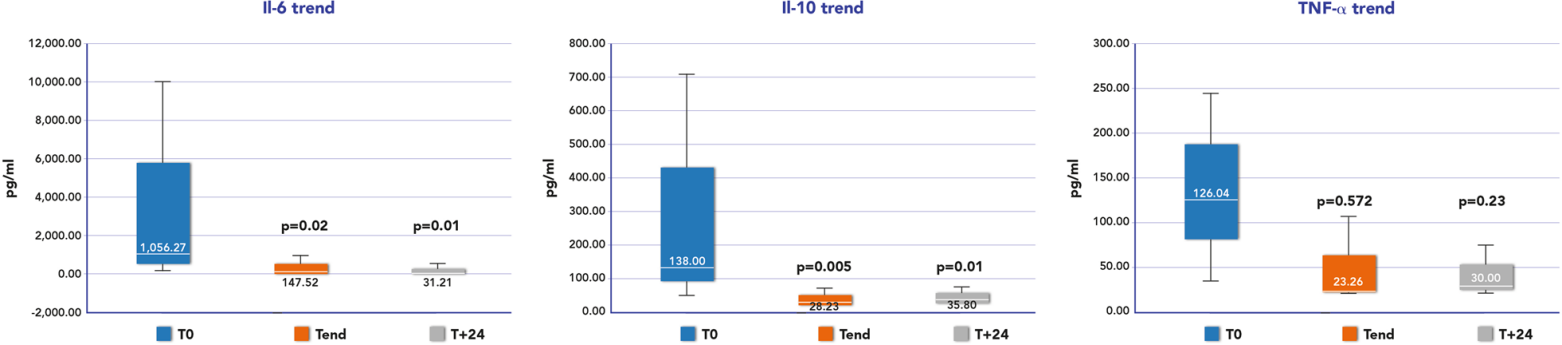
Box plots for time courses of cytokines Interleukin 6 (Il-6), Interleukin 10 (Il-10) and Tumor Necrosis Factor alpha (TNF-a) during blood purification with CytoSorb^®^ and Continuous Renal Replacement Therapy (CRRT). Data are presented as median and interquartile ranges. Timepoints: *t*_0_ = onset of hemoadsorption; *t*_end_ = end of hemoadsorption; *t*_+24h_ = 24 h after the end of treatment *t*_+24h_

Our findings show that hemodynamic improvements in our cohort of children with septic shock occurred in parallel with an immune-modulatory effect on cytokine hyperproduction during the blood purification treatment, based on CRRT and CytoSorb^®^. Secondly, evidence that 24 h after the end of hemoadsorption, no rebound effect was observed, suggests that the extracorporeal therapeutic effect could induce a substantial impact on immune-homeostasis. Finally, in our cohort we found no immunological patterns that would suggest immune-paralysis [4], or that hemoadsorption with CytoSorb^®^ and CRRT would impact cellular immune function, as suggested from the time course of HLA-DR expression and leukocyte count.

The main limitation of our study is the lack of a control group confirming irrevocably that the substantial reduction in the cytokine levels observed was associated with the adjuvant therapy and not the consequence of the natural course of the illness or potential effects of the standard therapy. Similarly, no definitive conclusion can be drawn between the impact of CytoSorb^®^ and of immune-dysfunction in our cohort. Furthermore, we did not measure pre- and post-cartridge cytokines levels as recently described by Jansen et al. showing in an experimental model of endotoxemia that CytoSorb^®^ hemoadsorption effectively attenuates circulating cytokine concentrations [5]. The transfer of this experimental model to every day clinical practice would allow cytokine removal efficacy to be shown in a real-life setting, but it requires adequate definition of the serum sampling protocol, which, in our setting, must inevitably be a compromise between the need for punctual and precise removal quantity definition, and effective blood conservation strategies within pediatric critical care.

Potential beneficial effects of hemoadsorption on leukocyte reprogramming [1] in pediatric septic shock patients with immune-dysfunction (4) should be investigated in larger cohorts with control groups and a longer follow up of immunological parameters.

### Supplementary Information

**Additional file 1: Fig. S1.** Upper section: box plots for time courses of HLA-DR percentage (HLA-DR %) and quantitative measurement with flow cytometry (HLA-DR MFI) during blood purification with CytoSorb® and Continuous Renal Replacement Therapy (CRRT). Data are presented as median and interquartile ranges. Timepoints: onset of hemoadsorption (D0), after 3 days (D3) and after 7 days (D7). Lower section: median and interquartile ranges of leukocytes at D0, D3 and D7.

## Data Availability

All data analyzed and discussed in the framework of this study are included in this published article and its online supplementary information. The corresponding author may provide specified analyses or fully de-identified parts of the dataset upon reasonable request.
