# Supplementary figures and images for: Potential correlation between hemodynamic improvement and an immune-modulation effect in pediatric patients with septic shock treated with renal replacement therapy and CytoSorb®: an insight from the PedCyto study
Source: Crit Care. 2024 Jan 17;28:25. doi: 10.1186/s13054-024-04802-9 (PMC10792820; doi:10.1186/s13054-024-04802-9)

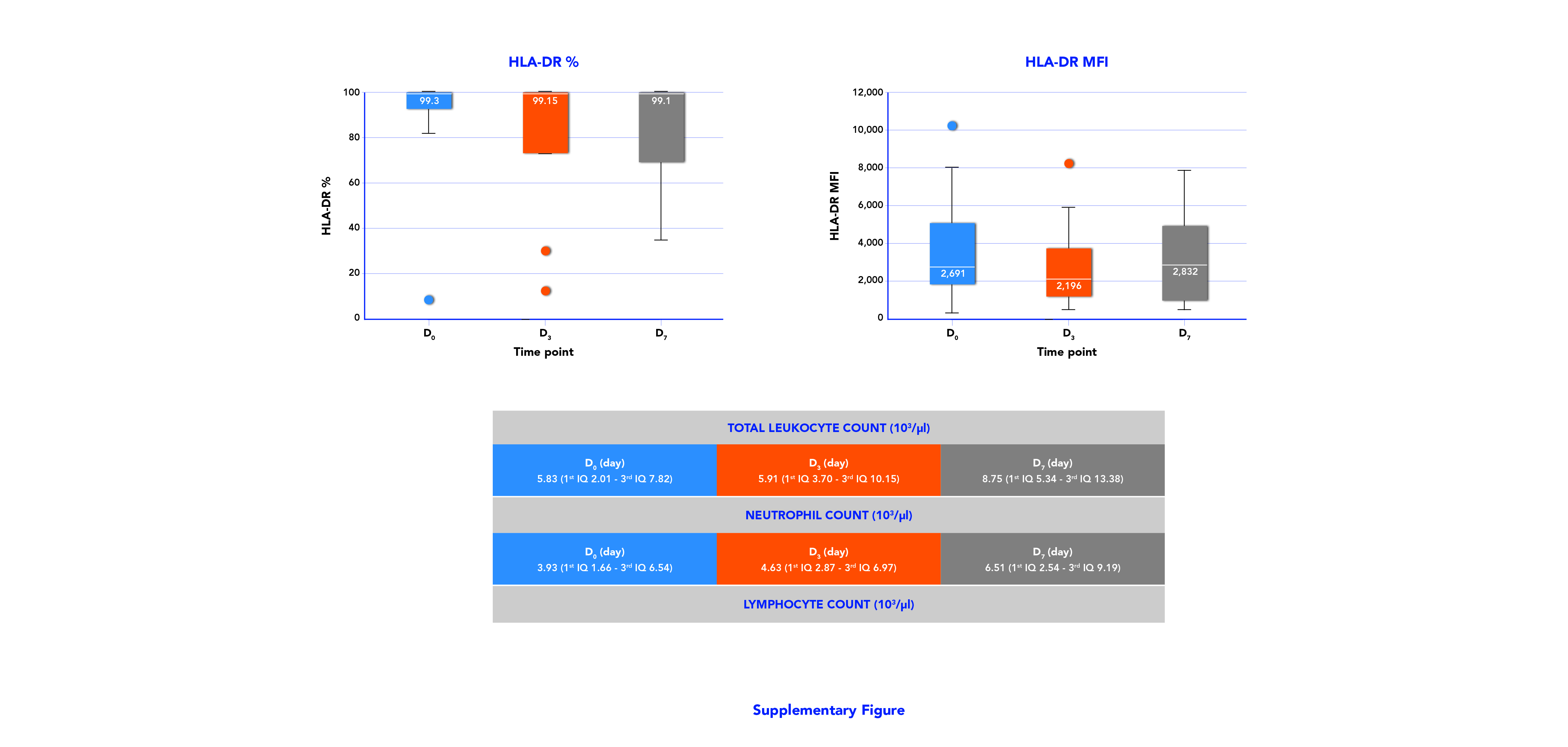

Supplement: Supplementary file 1 — Additional file 1: Fig. S1. Upper section: box plots for time courses of HLA-DR percentage (HLA-DR %) and quantitative measurement with flow cytometry (HLA-DR MFI) during blood purification with CytoSorb® and Continuous Renal Replacement Therapy (CRRT). Data are presented as median and interquartile ranges. Timepoints: onset of hemoadsorption (D0), after 3 days (D3) and after 7 days (D7). Lower section: median and interquartile ranges of leukocytes at D0, D3 and D7. [file 13054_2024_4802_MOESM1_ESM.jpg]
